# Virulence Factors of *Candida* spp. and Host Immune Response Important in the Pathogenesis of Vulvovaginal Candidiasis

**DOI:** 10.3390/ijms23115895

**Published:** 2022-05-24

**Authors:** Paulina Czechowicz, Joanna Nowicka, Grażyna Gościniak

**Affiliations:** Department of Microbiology, Faculty of Medicine, Wrocław Medical University, 50-368 Wrocław, Poland; joanna.nowicka@umw.edu.pl (J.N.); grazyna.gosciniak@umw.edu.pl (G.G.)

**Keywords:** *Candida*, vaginal infections, VVC, biofilm, microorganism-host interaction

## Abstract

Vulvovaginal candidiasis (VVC) is one of the most common types of vaginal infections in women around the world and is often underestimated by both patients and doctors. Research on the pathogenesis of fungal vaginal infections over the last 20 years has resulted in a closer understanding of the virulence factors involved in *Candida* epithelial invasion and their mechanisms of action. Recently, attention was drawn to the enormous complexity of the interaction between yeast-like fungi and host cells, as well as the level of complexity of the host’s response to infection and their impact on the course and treatment of VVC. Our work provides a broad description of already known and some new reports on *Candida* virulence factors (such as phenotypic switching or biofilm formation capacity) and their importance for tissue invasion in VVC. At the same time, we also focus on interactions with host cells and local innate immune mechanisms involved in the response to vaginal fungal invasion that are now considered equally important in this case. The presented review describes the most important aspects of the still unknown pathogenicity of *Candida* associated with vaginal infections.

## 1. Introduction

The first account of yeast fungus infections probably dates to the 5th century BC. The oldest documented reports indicating a clinical link between candidiasis and fungi were made in 1839. Since then, *Candida albicans* has been recognized as the main etiological factor of candidiasis and other diseases of the mucous membrane [1].

In the period between the discovery of fungi by the ancient Greeks to modern times, the view on the pathogenicity of these microorganisms has changed. It is now known that they are generally not pathogenic for people with a well-functioning immune system. However, in specific clinical situations they may become an etiological factor in severe, life-threatening infections [2]. It is worth noting that the incidence of infections caused by these microorganisms has been systematically increasing around the world, and mortality can reach 60–70% for some groups of patients [2,3,4]. All this makes fungal infections not only an epidemiological problem but a social one as well.

## 2. Overview of *Candida* spp.

### 2.1. Risk Factors Associated with Superficial and Invasive Candida Infections

Colonizing mucous membranes, mainly the oral cavity, intestines, vagina, and skin, fungi of the genus *Candida* are part of the human microbiota. As commensals, they do not cause infections in healthy people. They are considered opportunistic microorganisms which cause infections only in specific clinical situations and in the presence of favorable conditions. They mainly pose a threat to immunocompromised persons or patients hospitalized for a long time. People in these risk groups suffer from mucosal infections caused by switching commensal yeast-like fungi to pathogenic ones. Importantly, the risk factors described in detail below also create a predisposition to candidemia and other invasive candidiasis [5]. 

Factors predisposing to infection with fungi of the genus *Candida* can be classified into immunological and non-immunological [4]. The first group includes, among others, HIV infection. As the disease progresses, more than 90% of patients suffer from superficial fungal infections affecting mainly the oropharynx, primarily related to low levels of CD4+ lymphocytes, reduced activity of NK cells, and loss of T helper cells. The dominating species in this group of patients is *C. albicans*, but infections may also be caused by *Candida tropicalis, Candida krusei,* or *Candida parapsilosis* [4]. 

Immunosuppressive therapy, used, for example, in transplant patients, reduces the natural immune response, which makes the body more susceptible to all kinds of fungal infections, especially with *C. albicans*. The same is true for neoplastic diseases. The disease itself and the therapy used often lead to immunosuppression and disruption of the mechanisms of both humoral and cellular immune response [4]. The incidence of candidiasis among cancer patients treated in hospitals is higher compared to patients hospitalized for other causes [6]. 

A lowered level of leukocytes in the blood is an important risk factor, especially for patients with haematological disorders. It may result from the ongoing disease process, as well as from the therapy used (chemotherapy or antimicrobial treatment). It is worth adding that patients with chemotherapy-induced neutropenia are often in a serious condition that is additionally accompanied by the presence of other factors conducive to fungal infections (e.g., the use of broad-spectrum antibiotics and vascular catheters) [6]. 

Regarding non-immunological factors, it is worth mentioning disorders in the quantitative and qualitative composition of the microbiota, which promote the excessive multiplication of fungi. Diabetes and the associated high glycemia promote the colonization of fungi. In people with diabetes, much higher colonization of the oral mucosa with *Candida* fungi has been observed compared to the healthy population [3,4]. Reduced saliva flow or impaired neutrophil activity seem to be major factors here [3]. Hormonal disorders, especially in pregnant women, are also worth mentioning. Drugs that reduce the secretion of gastric juice or insufficient secretion of digestive enzymes promote the multiplication of fungi in the digestive tract. Inadequate diet, alcohol, lack of vitamins (especially vitamin A, vitamins B6, B12, and C) and microelements (selenium, zinc, iron), smoking, aging of the body, or chronic stress favor these types of infections [4,7]. Not without significance are also past surgeries, especially in the abdominal cavity or parenteral nutrition [8]. Patients with recurrent intestinal perforation or acute necrotizing pancreatitis have a higher risk of candidaemia [6]. The same applies to total parenteral nutrition—it increases the risk of candidiasis almost 4-fold [8]. The presence of a vascular catheter and its colonization may constitute the port of entry for the fungi to the bloodstream [6]. Past surgery, parenteral nutrition, or the use of endovascular catheters are all factors increasing the risk of (mainly systemic) infections [8].

Fungal infections often occur in extreme age groups: newborns, especially premature babies with low birth weight, and the elderly. Undoubtedly, the above is related to the functioning of the immune system—newborns do not have well-developed specific and nonspecific immune responses. In the case of older people, due to age, the immune system is no longer as efficient [9,10]. There may be additional factors in both groups. In the case of newborns, an important factor is the absence of microbiota in the gastrointestinal tract. A disruption in the process of acquiring a microbiota can lead to colonization by pathogenic microorganisms, including fungi. Hospitalization at an ICU, the use of vascular catheters, parenteral nutrition, and antibiotic therapy make them a group with a higher risk of infection [6,9]. It is worth adding that the risk of infection is higher for newborns with bodyweight of less than 1000 g than for newborns weighing more than 2500 g [6]. Similarly, in the case of the elderly, the use of immunosuppressive therapy, broad-spectrum antimicrobial drugs, or comorbidities such as diabetes often increase the risk of infections, especially in patients over 65 years of age [11].

The ability of fungi of the genus *Candida*, especially *C. albicans*, to colonize or infect various sites (tissues) in the human body depends on many pathogenic factors of these microorganisms. Some of them, especially those important in the context of inducing vulvovaginitis, will be discussed in more detail later in the paper. It may be worth emphasizing. However, morphological changes and phenotypic switching, expression of a number of adhesins and invasins on the cell surface, the ability to form a biofilm, or the secretion of hydrolytic enzymes are considered the most important pathogenicity factors of these microorganisms. They allow colonization, adhesion, invasion, and damage to the host tissues. Their ability to adapt to the changing pH of an environment, as well as efficient systems for obtaining nutrients [12,13], are also of great significance.

### 2.2. Most Commonly Isolated Species

*C. albicans* is the species most often responsible for infections. However, it is worth noting that in recent years there has been an increased incidence of isolation of species other than *C. albicans*: *Candida glabrata*, *C. tropicalis*, *C. parapsilosis*, *Candida dubliniensis*, *Candida guilliermondii*, *C. krusei*, or *Candida kefyr* [2,14]. Epidemiological data confirm this trend worldwide [14]. Recent years witnessed a particular increase in the incidence of candidiasis caused by rarely isolated *C. krusei* or *C. guilliermondii* [15].

The suspected causes of the change in the epidemiology of fungal infections are the frequent use of compounds with antifungal activity, both in the prevention and empirical treatment of infections. Abuse or improper use of antifungal drugs are most likely causes of reduced susceptibility or even resistance of fungi to antifungal drugs. Increasingly, species other than *C. albicans* are responsible for infections, and, importantly, they demonstrate a reduced susceptibility to antifungal drugs [15,16]. When analyzing the frequency of isolation of yeast-like fungi from various clinical materials, Taei et al. showed that among NCAC (Non-*Candida albicans Candida*) species, *C. glabrata* was most often isolated (it accounted for 76% of all NCACs). The next most isolated were *C. krusei* (6.6%), *C. kefyr* (5.7%), *C. parapsilosis* (4.9%), and *C. tropicalis* (2.4%). The least common (0.8%) were *C. dubliniensis*, *C. guilliermondii,* and *Candida famata*. *C. albicans* accounted for 38.5% of all isolated fungi of the genus *Candida* [14]. According to the authors of the aforementioned paper, the increase in the frequency of NCAC isolation is a result of better laboratory diagnostics as well as previous exposure to polyenes and azoles or of reduced immunity, which is often related to the therapy used, e.g., cytostatics, in transplant or cancer patients. Diabetes was a common factor conducive to NCAC infections [14]. Research by Das et al. showed a similar relationship. Of the 112 *Candida* fungi isolated from vaginal swabs, 58% were non-*C. albicans* species. Among them, *C. glabrata* (20%), *C. tropicalis* (19%), and *C. parapsilosis* (9%) were isolated most often. *C. albicans* was isolated in 42% of cases [17]. Despite *C. albicans* being the most commonly isolated, an increasing share of NCAC species and their dominance in complicated vulvovaginitis was observed. The authors explain the above primarily by prolonged antifungal treatment, diabetes, older age, and poor hygienic conditions. NCAC species were also shown to have a higher resistance to antifungal drugs compared to *C. albicans* [17]. Liu et al., who focused on the assessment of risk factors for NCAC candidiasis, showed that *C. albicans*, *C. tropicalis*, *C. glabrata*, *and C. parapsilosis* were most often isolated. The above species accounted for more than 96% of all isolated fungi of the genus *Candida*. NCAC and *C. albicans* were isolated in 53.5% and 46.5% of candidiasis cases, respectively. Among NCACs, *C. tropicalis* was most isolated, primarily from patients with haematological tumors. The authors have demonstrated a higher resistance among NCAC species, particularly of *C. tropicalis* isolates [18]. 

When discussing species other than *C. albicans*, *Candia auris* is worth mentioning. First isolated in Japan in 2009 from a patient with otitis, it has become a causative agent of invasive infections around the world. It is characterized by ease of spreading, resistance to antifungal agents and disinfectants, a wide range of pathogenic factors, and the fact that it causes severe infections with a high, estimated at even 72%, mortality rate [19,20,21]. Most often, it causes infections in patients who are artificially ventilated, hospitalized in the intensive care unit, catheterized, HIV-infected, diabetic and immunosuppressed. Other factors predisposing to *C. auris* infections include parenteral nutrition, previous surgery, or long-term use of antimicrobials [19,20,22]. *C. auris* is currently a global threat, causing severe epidemic outbreaks of invasive infections most often associated with medical care [21].

### 2.3. Infections Caused by Fungi of the Genus Candida

Among the infections caused by *Candida* spp. fungi, one can distinguish surface infections and systemic infections [12,19,20,23]. Surface infections are not generally life-threatening [12]. Among them, one can mention infections of the skin and nails, mucous membrane of the mouth, throat, esophagus, intestines, and vagina [12,24]. Systemic infections, on the other hand, are characterized by a severe course and pose a direct threat to human life. Systemic candidiasis is associated with high mortality, and neutropenia, as well as damage to the gastrointestinal mucosa, are quoted as the most common factors predisposing to this type of infection. Other factors include the use of central venous catheters or antibacterial therapy, most often with broad-spectrum agents [12]. 

Skin infections are most often surface infections, while infections involving the dermis and subcutaneous layer occur very rarely. The changes most often affect skin folds, e.g., inguinal folds, and often occur in overweight people. *Candida* spp. may also be the causative agent of paronychia or onychomycosis [23]. Oral candidiasis is mainly caused by *C. albicans* and most often occurs in people with impaired immune function. The most common risk factors are HIV infection, followed by wearing prostheses and braces, old age, xerostomia, or poor oral hygiene [12,23]. Esophageal candidiasis is most often caused by *C. albicans* and occurs primarily in people with an impaired immune system or with concomitant diseases, e.g., diabetes. Often, it is manifested by odynophagia and dysphagia, but it can also be completely asymptomatic [23]. Intestinal mycosis is most often superficial, but it can also occur with intestinal perforation. Fungal infections of the intestines are most often associated with inflammatory bowel disease [23].

According to statistical data, about 75% of women have experienced vulvovaginal candidiasis (VVC) at least once in their lifetime, and in about 5–8% of them, it has a recurrent character (occurs at least four times per year) [12]. The pathomechanism of VVC and the pathogenicity of *Candida* spp. fungi associated with vaginal infections will be discussed further in the paper.

Invasive infections are most commonly caused by *C. albicans*, *C. glabrata*, *C. tropicalis*, *C. parapsilosis*, and *C. kefyr* [23]. These fungi are some of the most common etiological factors of invasive infections, primarily in patients in intensive care units [25]. The entry of fungi into the bloodstream may result in the spread of fungi into tissues and organs [24]. It can cause, among others, meningitis, peritonitis, and abdominal infections, endocarditis, or infections affecting the patient’s bones and joints [23]. The above-mentioned risk factors for invasive infections also include old age, parenteral nutrition, cancer, and immunosuppressive treatment [25].

*Candida* spp. fungi are also causative agents of infections associated with a foreign element (biomaterial) introduced into the human body. In this context, the adhesive properties of fungi and their ability to form biofilm are important. The possibility of fungal colonization in hip replacements, vascular catheters, urinary catheters, endotracheal tubes, dental implants, artificial valves, pacemakers, or even contact lenses has been demonstrated [26,27]. Infections that occur with the formation of biofilm often have a chronic, recurrent character and are characterized by a high mortality rate of more than 40% [27]. Biofilm plays an important role, protecting the fungal cells against the immune mechanisms of the human body as well as against antifungal agents. The therapeutic options for infections with biofilm-forming organisms are extremely limited [27]. The formation of biofilm structures on the biomaterial may result in blood infection or systemic infection of tissues and organs [28].

## 3. Vulvovaginal Candidiasis

As noted earlier, vulvovaginal candidiasis affects many women around the world. After bacterial infections, it is the second most common type of vaginal infection [12,29,30,31]. The clinical picture may vary, but most often, its symptoms include itching of the vagina and vulva, soreness, burning, and abnormal vaginal discharge. Other symptoms may include difficulty urinating and pain during sexual intercourse [29]. Data in the literature show that the incidence of this type of infection partially depends on the age of the woman, which also determines sexual maturity, hormonal activity, and the state of the vaginal microbiota [29,30,32]. The most common factors predisposing to VVC are pregnancy, the luteal phase of the menstrual cycle, the use of broad-spectrum antibiotics, the use of intrauterine devices, estrogen replacement therapy, immunosuppression, diabetes, or mechanical factors, e.g., the use of tight, synthetic underwear [31,33,34]. 

Research by Zeng et al. showed that women under 40 years of age had a higher risk of vaginal mycosis than older women [29]. It is also rarely diagnosed in patients before puberty [33]. Vulvovaginal candidiasis occurs primarily in young women of childbearing age and rarely in premenopausal women [30]. Almost 55% of women before the age of 25 will experience a VVC episode [33]. Sexual activity and physiological and tissue changes that are caused by reproductive hormones that occur in women during this period of life, seem to be important here. They increase susceptibility to *Candida* spp. infection [29].

To a large extent, the above has to do with the activity of sex hormones during the reproductive period and with low levels of estrogen and progesterone during menopause. During reproductive age, high levels of estrogen induce the hypertrophy of the vaginal mucosal epithelium as well as the secretion of glycogen by vaginal epithelial cells. Its fermentation to lactic acid by *Lactobacillus*, the main component of the vaginal microbiota, leads to a significant decrease in pH, which prevents the development of other microorganisms. In addition to ensuring proper vaginal pH, *Lactobacillus* bacteria, primarily *Lactobacilluis crispatus*, *Lactobacillus gasseri*, *Lactobacillus jensenii*, and *Lactobacillus iners*, produce a number of substances, such as hydrogen peroxide or bacteriocins, which inhibit the multiplication of other microorganisms. They also compete for nutrients and receptors on vaginal epithelial cells with other microorganisms [30,32]. In menopausal women, a drop in estrogen levels and vaginal atrophy occurs, leading to a decrease in glycogen levels in vaginal epithelial cells and a neutral pH [32,34]. As Hoffmann points out, vulvovaginal candidiasis often occurs at lower pH values of vaginal secretions, which is the case for healthy women of childbearing age. Low hormone levels and vaginal atrophy are associated with a much lower risk of VVC in postmenopausal women [34]. It is generally accepted that a decrease in the level of lactobacilli or their complete absence predisposes women to VVC [30]. However, in his 2010 paper, McCelland showed that colonization of the vagina with *Lactobacillus* increases the risk of VVC by a factor of four [30,35]. Additionally, Ceccarani et al. showed that the vaginal microbiota of healthy women was dominated by *L. crispatus*. In a state of disease, the abundance of this species decreased while the number of *L. iners* increased significantly. The same relationship was also demonstrated by the mentioned authors for VVC. Additionally, high levels of glucose in the vagina of women with VVC were shown. On the one hand, glucose is a nutrient for *Candida* spp. However, on the other hand it promotes the expression of binding molecules in vaginal epithelial cells, thus increasing the adhesion of *Candida*. As the authors point out, high levels of glucose in the vagina may be associated with a reduction in the abundance of *L. crispatus* [36].

Data in the literature show that vaginal colonization with *Candida* spp. is greater in pregnant women (especially in the second and third trimester of pregnancy) than in non-pregnant women. The above state of affairs is most often explained by reduced cell-mediated immunity, high estrogen levels, and high glycogen levels, which promotes colonization with *Candida* spp. [37,38].

The diagnosis of vulvovaginal candidiasis is based on symptomatic vulvovaginitis with simultaneous isolation of *Candida* spp. from the clinical material (or from a vaginal swab) in the absence of any other etiology [30,39]. As mentioned earlier, about 75% of women will experience vulvovaginal candidiasis (VVC) at least once in their lives, and in about 5–8% of them, it will be recurrent (at least four VVC per year) [12]. Recurrent vulvovaginal candidiasis affects nearly 138 million women annually [39].

In the vast majority of cases, the etiological factor of VVC is *C. albicans*. However, more and more frequently, the causative agents are species other than *C. albicans*, e.g., *C. glabrata*, *C. parapsilosis*, *C. tropicalis* or *C. krusei* [39]. An increase in the isolation of NCAC species in recurrent and complicated VVC was observed [17,39]. It is commonly known that species other than *C. albicans* are characterized by greater virulence and resistance to antifungal agents, which often leads to therapeutic failures [39].

## 4. Pathogenicity of Fungi of the Genus *Candida* spp. Related to Vaginal Infections—Virulence Factors

The detailed pathomechanism of vulvovaginal candidiasis remains unexplained. On the one hand, it is linked to imbalances in the vaginal microbiota and, on the other hand, to an abnormal immune response of the mucous membrane to the presence of fungi of the genus *Candida* [40,41,42,43,44]. Among the factors potentially responsible for the evolution of asymptomatically colonizing yeast-like fungi into invasive pathogens and, as a result, for the development of symptomatic vaginal infection are both dependent on the host cells and many factors of fungal virulence [43,45]. The pathogenicity of *Candida* spp. in VVC is related to the following: adhesive properties, both in relation to abiotic surfaces and to mucous membranes; the synthesis and secretion of many hydrolytic enzymes, among others, hydrolases or phospholipases; the formation of biofilm structure; the ability to grow in extreme environmental conditions; reversible filamentation; phenotypic switching [42,43,45,46,47]. Disputes are ongoing to determine which of these factors is the most important in the development of VVC, with many authors pointing to its multifactorial pathogenesis. Thus, the key task seems to be to gain knowledge about the interactions between vaginal epithelium and the fungi of the genus *Candida* and about the mechanisms of cell invasion by these microorganisms.

### 4.1. Adhesion

The adhesion ability is unquestionably the key factor in the virulence of *Candida* spp., necessary for both the initiation of colonization and the development of infection [42,47,48]. *Candida* spp. cells are capable of adhering to biotic surfaces, as well as to various types of biomaterials [42,44,47,49]. Adhesion is also the first stage of forming a biofilm structure, allowing mature biofilm to be formed on both types of surfaces. In the context of the pathogenesis of VVC, the ability of *Candida* spp. to adhere to the surface of the mucous membrane of the vaginal epithelium is important. This process most likely determines the development of the infection. However, according to epidemiological data, a symptomatic vaginal infection may also be associated with the adhesion of fungal cells to the surface of intrauterine devices, with the formation of a biofilm structure on them and with the invasion of the surrounding tissues [47].

Adhesion is a complex and multifactorial process. The earliest stages of adhesion consist of the interaction of yeast cells with the colonized surface by means of non-specific interactions associated primarily with cell hydrophobicity and the occurrence of electrostatic forces [8]. The degree of adhesion depends on the type and species of the microorganism, the response of host cells to the presence of a given strain, and the properties of the surface structure and its composition [42]. The surfaces of *C. albicans* and *C. glabrata* cells show a similar degree of hydrophobicity. In the case of *C. glabrata*, the degree of hydrophobicity appears to be more stable under changing environmental conditions, which is indicated as the reason for the twice higher tendency of these strains to adhere to acrylic surfaces of prostheses in comparison to *C. albicans* [42,50].

Further phases of adhesion are associated with the presence of adhesins—specific adhesive proteins [8,42,44,47,48]. The expression of adhesive proteins occurs in the fungal cell wall. They mediate adhesion by recognizing and binding their specific ligands (laminin, fibronectin, collagen, fibrinogen, vitronectin or complement proteins) on the surface of the host cells [8,47]. Different adhesive proteins often demonstrate various expression on blastospores and hyphae in different species and have different mechanisms of action [48]. 

One of the adhesin groups characterized in most detail are Als proteins, encoded by a large family of *ALS* (agglutinin-like sequence) genes [8,42,47,48]. It is a group of eight proteins (Als1–7 and Als9) bound to glycosylphosphatidylinositol (GPI). Each consists of three domains: N-terminal, having a region binding to a specific substrate, centrally located tandem repeat sequences, and C-terminal, containing the GPI anchor sequence. *ALS* genes are present in *C. albicans* strains, and they were thought to be specific to this species. However, a family of five *ALS* genes in *C. parapsilosis* and three *ALS* genes in *C. tropicalis* was discovered. Additionally, Als3 homologues were found in *C. dubliniensis*, *C. guilliermondii* and in *C. lusitaniae* [8,42,48].

Two approaches are currently developed and used to assess the role of Als in adhesion. These include studying the expression of individual Als characteristics for *C. albicans* in *Saccharomyces cerevisiae* which do not demonstrate adhesive properties, and determining the degree of adhesion in mutants in which both copies of a given *ALS* gene have been removed. What is surprising is a quite frequent lack of compatibility of the results obtained using individual methods. Increased expression of Als5 and Als6 in *C. albicans* correlated with an increase in the degree of adhesion, while gene deletion for these proteins also resulted in increased adhesion. The authors of the tests indicate that perhaps deletion results in a compensatory increase in gene expression for other adhesins and hence the cumulative increase in the adhesion of such strains [48]. Confirmation of this hypothesis requires more detailed research, as does the determination of the exact role of individual Als proteins in adhesion [42,44,47,48].

Another group of adhesive proteins are epithelial adhesins (Epa), whose *EPA* gene expression is induced by the presence of nicotinic acid. Epa1, with the properties of lectin dependent on calcium ions, is considered the most important representative of this group of proteins [42,51]. Interestingly, it has been shown that gene deletion only for Epa1 results in an almost complete loss of adhesive abilities in a given strain [42]. Epa are associated with the adhesion of *C. glabrata* strains to epithelial cells and biomaterials. Unfortunately, the mechanism of their action and their role in adhesion are not very well characterized and require more in-depth analysis [8,42,51].

Another crucial factor in the adhesion of fungal cells to the epithelium is Hwp1 (hyphal wall protein 1)—the protein of the cell wall characteristic of the hyphae form [8,41,44,48]. Hwp1 is one of the key virulence factors of yeast-like fungi occurring as hyphae, displaying a unique mechanism of action. Hwp1 has a GPI anchor domain like adhesive Als and Epa, while its N-terminal sequence is a substrate for transglutaminases binding to epithelial cells. Thanks to these enzymes, Hwp1 (and the entire fungal cell) binds to various proteins on the surface of the host epithelium. It has also been proven that this adhesin can bind other adhesion proteins, e.g., Als1 and Als3 *C. albicans*, conditioning the auto-adhesion of blastospores-hyphae and hyphae-hyphae, a key interaction within the biofilm structure. Strains with both copies of the gene deleted for Hwp1 showed a drastically reduced virulence in a mouse model of oropharyngeal candidiasis, which indicated that this adhesin is a key virulence factor in *Candida* spp. [48]. 

Fungi of the genus *Candida* express many other adhesion proteins and non-protein factors with similar properties, such as Eap1, Iff4, Mp65, Ecm33, Utr2, Int1, or Mnt1 [8,48]. However, because they have not yet been fully characterized and their mechanism of action is unknown, they will not be discussed in more detail. Therefore, it is currently impossible to determine which of them and how exactly they interact with the mucous membrane of the vagina and what role they play in the pathogenesis of VVC.

### 4.2. Hydrolytic Enzymes

The production of hydrolytic enzymes serves *Candida* spp. strains both to obtain nutrients by digesting various molecules, and it is also a virulence factor. Enzymes facilitate tissue invasion and inactivation of the components of the host immune system [52]. Although most authors are predominantly interested in various hydrolases secreted extracellularly by yeast-like fungi, *Candida* strains also have the ability to produce endopeptidases [53]. The most important groups of hydrolytic enzymes include proteases, hemolysins, as well as lipases, and phospholipases [8,42,47,53,54,55,56].

Secreted aspartyl proteases (Sap) are the *Candida* enzyme group that has been characterized most extensively and in most detail. They are encoded by a large family of *SAP* genes. To date, ten genes have been identified in *C. albicans* (*SAP1–10*), three in *C. parapsilosis* (*SAPP1–3*), at least four in *C. tropicalis* (*SAPT1–4*), and more recently, seven genes in *C. dubliniensis* [24,42,47,50,54]. *C. glabrata* is the only one without these genes, and it is capable of producing 11 yapsins, proteolytic enzymes encoded by a cluster of 11 *YPS* genes. They are highly similar to Sap and *S. cerevisiae* yapsins, anchored on the cell surface, of fundamental importance in maintaining cell wall integrity, adhesion, and survival in macrophages [8,53,54]. Sap is associated with the formation of hyphae, increased adhesion, and phenotypic variability, so they facilitate colonization and invasion of host tissues. In addition, they can degrade defense proteins produced in response to the presence of fungi: immunoglobulins, α-macroglobulin, lactoperoxidase, collagen, creatine, mucin, fibronectin, or complement proteins (C3b, C4b, C5) [24,45,52,53,57]. Conventionally, they were classified into three groups based on substrate specificity and amino acid sequence homology: Sap1–3 with sequence homology of up to 67% and much greater importance in mucous membrane infections, Sap4–6 with sequence homology of nearly 90%, associated with systemic candidiasis, and Sap7 (and, according to some authors, also Sap10), which are the most distinguishable in this family of enzymes, whose functions have not been fully identified [53,57,58]. At the same time, Sap1–3 are produced by blastospores, while the expression of *SAP4–6* genes occurs mainly at the ends of hyphae [8,24,53]. Interestingly, contrary to their name, Sap9 and Sap10 are classified as endopeptidases and are anchored with GPI to the cell surface. Their functions are related, like *C. glabrata* and *S. cerevisiae* yapsins, to the maintenance of cell wall integrity and adhesion [53,54,58]. Knowledge about secretory proteases of fungi of the genus *Candida* is constantly expanding, and given the multitude of available in-depth studies on their structure and function as well as detection methods, the authors of this study will focus only on the potential role of Sap in the pathomechanism of vaginal mucosa infection [8,24,42,47,52,53,54,58,59].

Unlike other proteases, Sap have proteolytic activity only in an acidic environment, with a pH of four at most [47]. The fact that the normal pH value in the vaginal lumen is about four is the basis for the assumption that these enzymes may be one of the key virulence factors leading to the development of full-blown VVC. As already mentioned above, secretory aspartyl proteases possess wide substrate specificity. They can degrade many free and host cell-bound proteins that disrupt colonization and invasion by yeast-like fungi [56]. What seems to be the most important is the destruction by Sap of various compounds on the mucous membrane and the disturbance of homeostasis prevailing in this place, as well as the recognition and hydrolysis of inflammatory mediators produced in response [41,43]. The last two to three decades witnessed an abundance of studies aiming to determine whether *SAP* gene expression varies depending on the infection site. Such studies could explain the pathomechanism of various fungal infections by identifying specific enzymes that are crucial for developing candidiasis of a given type. This would make it possible to design experiments looking for ways to inhibit these Sap as part of a new antifungal therapy. Unfortunately, so far, the results of the above experiments have often been contradictory, and they failed to provide sufficiently reliable evidence documenting the role of individual Sap in various types of candidiasis. 

When discussing mucosal infections caused by *Candida* spp., the most frequent comparison is between oral candidiasis and vaginal candidiasis. A group of authors has indicated that aspartyl proteases can be found in the discharge of only sick women with VVC, as opposed to asymptomatic carriers [56,60]. However, most studies demonstrate that all described Sap can be isolated from *Candida*-containing samples taken both from patients and from only colonized individuals [58]. A study of *SAP* gene expression levels showed that Sap1–3 most likely play a key role in both oropharyngeal and VVC infections [24,53,58]. Mutants lacking all three genes caused significantly less damage to the mucosal epithelium and reduced the activity of pro-inflammatory cytokines in these sites. Confirmation was obtained in studies conducted on mouse models and on strains isolated from infected humans. However, experiments using reconstituted human epithelium (RHE) cells contradict these reports [53]. Sap2 was the dominant protease of *C. albicans* isolated from oral and vaginal infections and colonizing these areas [24,58]. Sap1 (dominant) and Sap3—both closely related to the phenomenon of phenotypic switching—turned out to be much less common proteases in asymptomatic carriers. Comparing the results obtained during the study and literature data, the authors pointed to the colonization and infection of the vaginal epithelium as the likely main role of Sap3. In combination with Sap1, on the other hand, this role is to enable adaptation to changing environmental conditions and survival in the lumen of the vagina [58].

Next to Sap, an often-mentioned group of hydrolases are phospholipases. They hydrolyze the phospholipids of cell membranes, contributing to the induction of host cell lysis and further penetration into the tissue. They are directly related to the progression of fungal infection [8,42,45,47,50,52,53,55]. There are four classes of phospholipases, depending on the ester bond they cleave: A, B, C, and D. Almost all *Candida* spp. strains are capable of synthesizing various phospholipases belonging to all these classes. *C. albicans* produces much higher amounts of them than the representatives of the NCAC group [8,42,47,50,52,58]. The literature provides much less information about this group of enzymes, although they are an object of interest to an increasing number of researchers. Unfortunately, the few experiments looking for gene expression patterns for individual phospholipases, such as *PLB1*, *PLB2*, *PLC1*, or *PLD1*, did not allow us to draw valid conclusions about their role in the pathogenesis of mucous membrane infections [47,53,58]. 

Regarding yeast-like lipases produced by fungi, the literature also provides only a limited amount of information. They are involved in the hydrolysis and synthesis of triacylglycerols. Most likely, they increase adhesion and penetration of host tissues, as well as survival in macrophages [42,47,52,53,59]. There are currently ten known *LIP* genes in *C. albicans*, 2 in *C. parapsilosis,* and one lipase sequence in *C. tropicalis* [42,50,53,59]. However, preliminary reports indicate that the role of lipases in mucous membrane infections is limited and rather insignificant in VVC [53]. 

Among other enzymes acting as virulence factors of *Candida* spp. are hemolysins which are necessary for iron acquisition and survival in the host organism [41,42,45,47,50] and esterase with probable cytotoxic effects [55]. However, they are discussed sporadically, and there is a lack of detailed information about their structure and function or about their role in the development of individual types of candidiasis.

The experiments conducted using various research models and enzyme detection methods did not show any correlation between specific hydrolytic enzymes and infection, including VVC. It is highly likely that gene expression and their levels for different *Candida* enzymes change not only depending on the site of infectionbut also during disease progression, depending on its severity [58].

### 4.3. Adaptability to Changing Environmental Conditions, Dimorphism and Phenotypic Switching

For many years, it was thought that one of the factors distinguishing *C. albicans* from other *Candida* species is the ability of these strains to grow in various morphological forms. This ability is called dimorphism, where attention is drawn to the possibility of *C. albicans* being present in the form of blastospores or blastoconidia (terms used interchangeably) and filaments. Equally often, one can find the term polymorphic growth in connection with the occurrence of four forms: blastospores, germ tubes, pseudo-hyphae, and true hyphae [8,24,42,44,50,59,60,61,62,63,64]. It has been confirmed that *C. dubliniensis* is also a truly polymorphic fungus [24,42,50,59,62,65]. At the same time, the possibility of forming pseudo-hyphae by *C. parapsilosis* and *C. tropicalis* is described [42,50,66]. An example of a *Candida* species lacking this ability is *C. glabrata*, which grows only in the form of extremely fine blastospores [42,50]. 

The phenomenon of dimorphism is closely related to the adaptability of *Candida* spp. to the surrounding microenvironment. The response to external signals, consisting in changing the morphology of fungal cells, is possible due to phenotypic switching. Typical examples include the ability of the described strains to adapt to changes in temperature, pH, oxygen availability, lack of nutrients, or the presence of serum by inducing filament growth. An important feature of phenotypic switching is its reversibility and the possibility of returning from the hyphae form to growing blastoconidia [8,60,61,63,64,66].

Studies show that the form of blastospores or yeast (Y form) is associated primarily with the colonization and spread of *Candida* in the host organism. Hyphae (H form) is considered invasive and capable of adhesion, secretion of proteolytic enzymes, and tissue destruction [8,24,43,56,60,61,62,63,64,66]. In addition, unlike blastospores, filaments have the ability to survive in macrophages and show reduced susceptibility or resistance to antifungal agents active against Y forms [50,60,64,65,66]. In this way, the importance of the phenomenon of phenotypic switching as a virulence factor with a significant role in infection is proved. The hypothesis that the presence of hyphae, and thus phenotypic switching, is required by *Candida* spp. for its virulence and for the development of infection is questioned [59,60,61,63,64]. Difficulties in confirming or denying this hypothesis concern the way in which the role of individual phenotypes in candidiasis is studied. In experiments on animal models, mainly mouse and rat models, comparisons are made between the virulence of wild strains and of mutants with the deletions of specific genes involved in changing the morphology from blastospores to filaments and vice versa. When conducting experiments in this way, two important issues should be kept in mind: the different pathogenesis of fungal infections in laboratory animals and the enormous complexity of molecular processes involved in phenotypic switching. The most common animal model of candidiasis is the mouse model of disseminated infection, in which the pathogen is applied directly into the caudal vein. Obviously, such an experiment cannot reflect the processes leading to the development of mucosal infection and endogenous infections. It does not provide sufficiently reliable information about the interactions of the Y and H forms with the epithelium [60,64]. Intensive genetic studies over the past two decades have provided a wealth of extremely valuable information on the regulation and causes of morphological and phenotypic changes in fungi of the genus *Candida*, although the authors point out that there is still more to be discovered than is known. The described phenomena involve both transcription factors promoting growth in the form of hyphae (such as Efg1, Cph1, Cph2, Tec1, Czf1, Ndt8, and Rim101) and negative hyphae-specific regulators (e.g., Tup1, Nrg1, Rfg1), as well as entire signal transduction pathways. At the same time, they are also involved in the regulation of other factors and processes related to other virulence factors of *Candida*. For this reason, it is not possible to distinguish reliably enough whether the reduced virulence of the mutant *C. albicans* with gene deletion for Efg1 and Cph1 (which makes these strains unable to form filaments) is associated with the absence of a hyphae form. The reason may also be the reduced gene expression for the cell wall proteins—adhesins and Sap—which are also affected by the aforementioned Efg1 and Cph1 and their activation pathways [24,59,61,64]. Thus, the very definition of dimorphism and phenotypic switching as virulence factors with a specific role in developing candidiasis causes many controversies. 

In the context of VVC of *Candida* etiology, the percentage of asymptomatic carriers of these microorganisms in the vagina should be taken into account. The above means that these fungi are tolerated by the immune system of the carrier women. *Candida* spp. is called commensal flora in such cases, although it is unclear whether the host organism benefits from its presence in this site [60,64]. Despite only scarce reports on the role of hyphae and phenotypic switching in VVC, the authors indicate quite unequivocally that in the case of samples isolated from patients with an active infection, only filament forms are present, while in asymptomatic carriers, there are only blastopores. There are reports that also in in vitro cultures of VVC-isolated strains and systemic infections, the phenotypic switching level was higher than in healthy, only colonized subjects [43,56,59,60,63,64]. It is believed today that blastospores adhere to the vaginal epithelium and only then abruptly switch into hyphae—similarly to the development of infections in the oral cavity and gastrointestinal tract. Filaments are not tolerated by the immune system and induce a specific inflammatory response. Two ways of mucosal invasion are possible: by induction of endocytosis through hyphae (a mechanism not fully understood) and by active penetration into the tissue. In the oral cavity environment, both pathways have been observed, with endocytosis dominating initially. As regards GI candidiasis, only active penetration of the mucous membrane occurs [64]. Unfortunately, no similar observations have yet been made for VVC. The H-form shows the activity of more virulence factors than blastospores, such as enzymes from the Sap family, with an undetermined role in the pathogenesis of VVC. Recently, it has also been pointed out that higher filament virulence is associated with factors that allow disruption and avoidance of the host’s immune response rather than with the expression of virulence factors [60]. Some authors explicitly suggest that the role of hyphae formation, while undoubtedly significant, is not crucial in the development of VVC [63].

Phenotypic switching enabling *Candida* fungi to grow in the form of blastoconidia and filaments is certainly not irrelevant for the symptomatic development of VVC. However, a detailed determination of the role of individual growth forms requires more thorough research and careful analysis, also taking into account other virulence factors associated with hyphae as well as molecular pathways involved in their regulation.

### 4.4. Biofilm

Biofilm is defined as a highly heterogeneous, multicellular, and multilayered three-dimensional structure in which cells are surrounded by an extracellular matrix (ECM), specialize in performing specific functions, and communicate with each other [8,42,43,44,46,47,49,50,67,68,69,70]. The great interest of researchers in biofilm structure is primarily related to its clinical significance—biofilm is highly resistant to antimicrobials and the host defense mechanisms [8,41,42,44,46,47,68,70,71]. At the same time, it is clearly indicated that it is the preferred and widespread form of growth of most microorganisms [44,47,50]. Hence, research on both the pathomechanism of various infections and their treatment must undoubtedly consider the biofilm-forming ability of the analyzed microorganisms.

Both bacteria and fungi, including *Candida* spp., can produce biofilm. Increasingly frequently, multi-species and mixed (bacterial-fungal) biofilms are studied [72,73,74]. They are widely described in the literature; hence their detailed characteristics will not be presented in this paper. 

Among yeast-like fungi of the genus *Candida*, almost all clinically relevant species form biofilm. Classically, the process of forming this structure is divided into four phases: early (adhesion), intermediate (multiplication, filamentation, and ECM production), maturation, and spreading (dispersion) [8,44,69,71,75]. The development of individual phases, including their duration and the architecture of mature biofilm, depend on *Candida* species and on environmental conditions, e.g., the surface to which the cells adhere [8,41,42,69,71]. Nevertheless, the general basic structure of fungal biofilm and its characteristics always remain similar. Importantly, the results of observation of biofilm structure and its formation obtained in vitro on biomaterials have already been confirmed in in vivo and ex vivo animal models, including biotic surfaces, primarily the mucous membrane (vaginal, among others) [41,44,67,69,70,75].

Basically, all virulence factors of *Candida* spp. discussed in this chapter are to a higher or lower extent related to biofilm formation (Table 1). Adhesion is the first and decisive stage initiating the development of biofilm in a given microenvironment. Many authors treat biofilm as a consequence of the existence of adhesion [8,41,44,47,50,67,75]. *Candida* spp. forms biofilm composed of blastospores and hyphae cells which serve as a scaffold for the entire structure and provide many proteins, such as the already mentioned adhesins [8,42,69,71,75]. Additionally, dispersion is possible owing to dimorphism and phenotypic switching, and blastospores detaching from the surface of mature biofilm show, among others, a higher tendency for adhesion [71,75]. Once again, attention should be paid to the multitude of complex dependencies and interactions of individual virulence factors and their components, as well as molecular mechanisms underlying their functioning. They are a significant obstacle for researchers in their efforts to define and isolate specific factors related to the pathomechanism of candidiasis.

In the case of VVC, the hypothesis regarding the key role of biofilm in the development of full-blown infection and the transition of the commensal form of *Candida* spp. into pathogenic form is associated with two main observations. First, routine microbiological diagnostics, carried out using classical methods of identification and drug susceptibility testing (in vitro), most often indicates the susceptibility of yeast-like fungi isolated from the vagina to conventionally used antimycotics—mainly fluconazole. Meanwhile, clinicians more and more often observe therapy failures and recurrences of infections. The existence of biofilm in vaginal mucosa, a structure highly resistant and impermeable to most antimicrobials, could explain the ineffectiveness of antifungal drugs, as well as constitute a reservoir of persister cells and be the cause of VVC recurrence [40,41,45,47,49,70,72,76]. Secondly, in the case of the most common type of infection in the vagina, BV (bacterial vaginosis), it has already been proven that the phenomenon of biofilm formation is important for the development of infection and is its underlying cause. Hence the suspicion that a similar pathomechanism could underlie fungal vulvovaginitis [47,67,70,77]. A similar situation occurs in the presence of biomaterials in the vaginal environment, e.g., an intrauterine device. In this case, it is already clear that biofilm is the basic cause and source of fungal infection [47,78]. The above arguments are relatively often cited as evidence for the role of biofilm in the pathogenesis of VVC and RVVC [72]. The above produces further questions for researchers—is biofilm necessary for vaginal colonization and the development of candidiasis? Is it the cause of the transition of saprophytic fungi of the genus *Candida* into pathogens in the vaginal environment? Does the growth of fungi in the form of biofilm affect the host’s immune response? [41,67,69,70] These questions remain unanswered. To our knowledge, the only report so far focusing on the observation of the *Candida* biofilm directly on the vaginal epithelium contradicts the hypothesis of the key role of this structure in the development of VVC, arousing skepticism in the scientific community about such a high interest in biofilm in the context of vaginal candidiasis [40,79]. 

Even if it is not the source of infection and does not determine the pathogenesis of infection, biofilm in VVC and RVVC remains an important factor in the virulence of fungi of the genus *Candida*. However, the significance of this phenomenon remains a hypothesis that requires many further in-depth studies on the interaction of fungi with the surface of the vaginal mucosa.

## 5. Pathogenicity of Fungi of the Genus *Candida* spp. Associated with Vaginal Infections—Host Immune Response

The role and significance of specific virulence factors of *Candida* are not the only factors that have remained unknown until today. After many years of intensive research on the immune mechanisms involved in the pathogenesis of VVC, there is still more to be discovered than is known. Nevertheless, especially in the last few years, several factors, and mechanisms potentially crucial for the development of fungal infection in the vagina have been identified, related to the host’s response to the presence of *Candida*.

**Figure 1:**
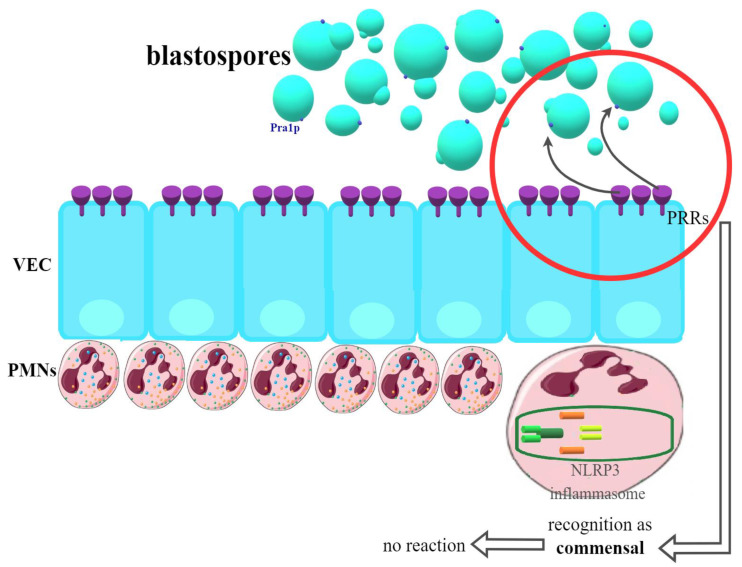
Recognition of *Candida* blastospores by VEC (vaginal epithelial cells) in women not susceptible to VVC (vulvovaginal candidiasis). PRRs (pattern recognition receptors) classify blastospores as commensals—no immunological reaction is provided.

It turned out relatively quickly that the acquired cellular or humoral immune response plays no role in VVC. Similarly, searches for the potentially protective role of innate immune components, such as macrophage or dendritic cell activity, did not reveal their effect on the vagina [43,80,81,82]. What has now been proven, vaginal epithelial cells (VEC) should be considered the first line of the host’s defense. On their surface, there are so-called PRRs (pattern recognition receptors) responsible for the recognition of *Candida*. Many authors emphasize that it is VEC that has the ability to distinguish and determine the fungi present on the vaginal mucosa as commensal or as pathogenic [43,80,81,83,84]. The most frequently cited hypothesis indicates the fungistatic action of epithelial cells, significantly limiting the development of infection and promoting the state of asymptomatic commensalism. The mechanisms underlying this phenomenon remain unknown. Annexin A1 can probably play a role here. The above way of VEC response predominates in women who are not susceptible to fungal infection, most often without a history of VVC/RVVC, in whom there is no further activation of the immune system [80,82]. This possibility is presented in Figure 1.

**Figure 2:**
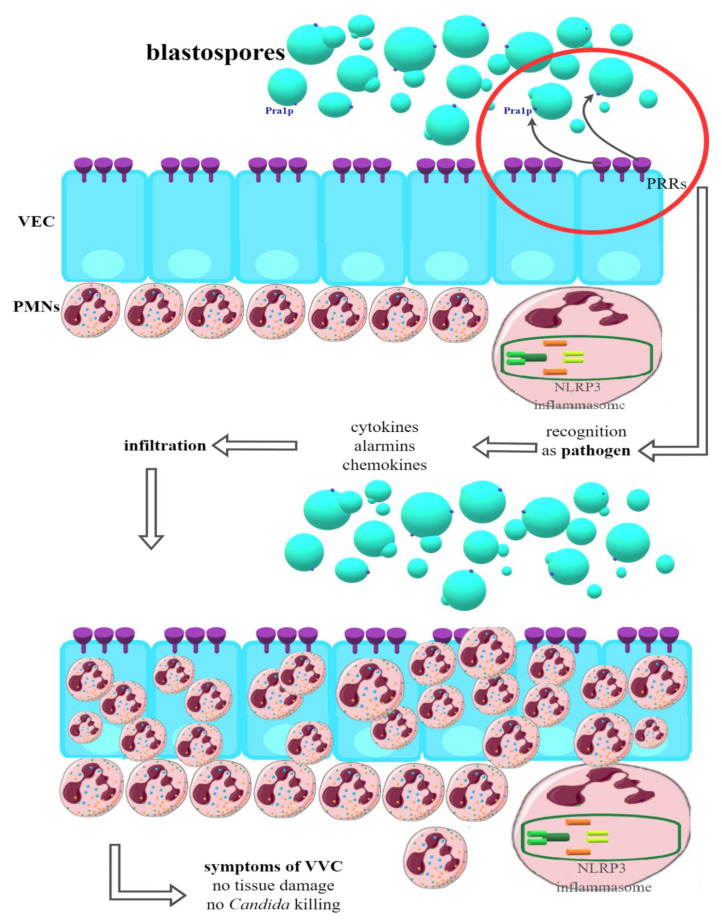
Recognition of *Candida* blastospores by VEC (vaginal epithelial cells) in women susceptible to VVC (vulvovaginal candidiasis). PRRs (pattern recognition receptors) classify blastospores as pathogens—production of proinflammatory cytokines, chemokines and alarmins is started. PMNs (polimorfonuclears) infiltrate VECs but are unable to kill *Candida* or directly damaging tissue. This state relates to clinical symptoms of VVC (detailed mechanism still unknown).

In women susceptible to infection (Figure 2), PRRs on the VEC surface recognize the components of the *Candida* cell wall and, in response, begin the production of a number of pro-inflammatory cytokines and alarmins, which in turn have a chemotactic effect on the PMN (polymorphonuclear) neutrophils located in the tissue stroma. Strong infiltration of vaginal tissue by PMN has been repeatedly shown in women manifesting clinical symptoms of VVC. It has been possible to clearly demonstrate that it is the immune response associated with PMN that is the cause of the symptoms of infection. In this way, one of the strongest pieces of evidence for the immunopathological origin of vulvovaginal candidiasis was provided, with VVC being an infection resulting not so much from the presence of the pathogen but the massive inflammatory response of the host organism and the resulting damage [43,80,81,84,85]. 

PMN cells migrating to the site of infection in response to the signals sent are not capable of effectively reducing or eliminating fungal cells. It has also been confirmed that they are not responsible for the damage to vaginal tissue associated with the infection that is observed during VVC [80,81]. Therefore, it is clear that the mechanism described above cannot be the only one underlying the infection in question. The virulence factors of fungi of the genus *Candida* and the resulting inflammatory response are also crucial. The most important in the immune context now seems to be phenotypic switching and hyphae growth. One of the most important factors associated with hyphae and identified recently is candidalysin, a toxin with lytic activity encoded by the *ECE1* (extent of cell elongation 1) gene. It works in two ways—on the one hand, it has the ability to activate the NLRP3 inflammasome; on the other hand, it causes damage to vaginal tissue directly [80,81,85]. NLRP3 is an intracellular receptor complex that performs a protective function in most types of candidiasis. In the vagina, however, after its oligomerization, caspase-1 and a further signal path are activated, because of which IL-1β, the main effector of inflammation, as well as alarmin S100A8, are produced and released. As a result, large amounts of many different pro-inflammatory cytokines, chemokines, and alarmins are released, which are also responsible for the recruitment of PMN to the site of infection [43,60,80,81,84,85,86]. Since PMNs are not able to effectively kill fungal cells in this case, the next step is their death and granular release, which, in positive feedback, causes further cytokine release and chemotaxis of subsequent PMNs, intensifying the inflammation [80,81]. In addition to candidalysin, other hyphae-related factors are also indicated that could act similarly, such as some Sap (although their role remains controversial), Als, lipases, or SAA3, activating NLRP3 [60,80,81,85,86]. 

**Figure 3:**
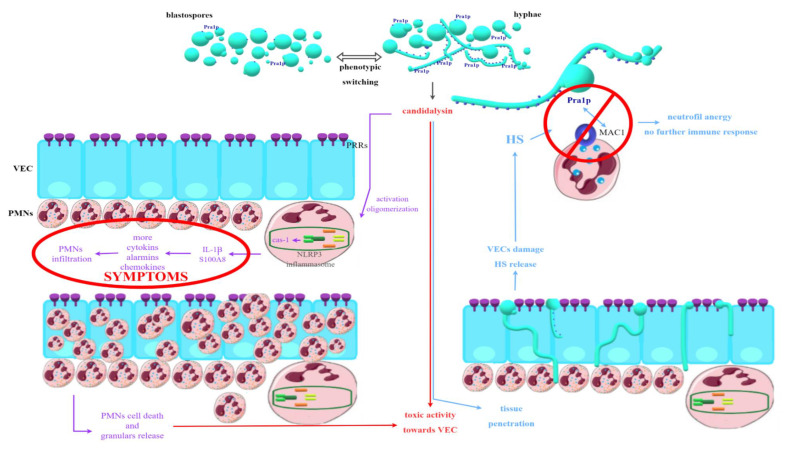
Hypothetical explanation of processes underlying tissue damage and clinical symptoms during VVC (vulvovaginal candidiasis) associated with production and release of candidalysin.

Candidalysin also causes direct damage to the VEC—it is now considered a key factor responsible for this effect. Experiments were carried out, which proved that the mere presence of hyphae (in the absence of toxin-producing ability) does not cause damage to the vaginal tissues during *Candida* infection. Therefore, it is highly probable that this peptide is the main factor behind the invasiveness of the hyphae form of yeast-like fungi and their ability to penetrate deep into the tissues [80,81,84]. In addition to its obvious clinical significance, VEC damage is also associated with yet another relatively recently discovered factor. It has been proven that VEC damage involves the release of, among others, heparan sulfate (HS). Observations confirmed that HS might be responsible for the so-called neutrophil anergy or inability to produce an immune response to an immunogen. The antifungal activity of PMN is conditioned by the specific recognition of fungal cells, which occurs through Mac1 present on neutrophils, binding to Pra1p (pH-regulated antigen 1 protein) in *Candida*. It is worth mentioning that this protein is present in a much higher density in hyphae cells than in blastospores. In other candidiasis types, Mac1 activation promotes the killing of fungal cells by creating so-called NETs (neutrophil extracellular traps). In VVC, this phenomenon does not occur, and it has been proven that HS acts as a competitive ligand for Mac1, blocking the target Pra1p binding point, which prevents the killing of *Candida* and is most likely responsible for the anergy phenomenon. Recent reports also focus on the role of estrogens in the development of infection. Their large amount also causes an increase in the release of HS and thus may contribute to additional impairment of cidal functions of PMNs. A possible role of estrogens in the direct activation of the NLRP3 inflammasome is also indicated [80,81,86]. The described hypothesis is presented in Figure 3.

The above interactions are only the first step in identifying key immune factors relevant to VVC immunopathogenesis. Researchers are currently interested in so many other immune components and mechanisms potentially involved in the host response to the presence of *Candida* on the vaginal mucosa that an attempt at listing them is far beyond the scope of this paper. However, it is worth emphasizing that there is no longer any doubt that for the development of symptomatic vaginal candidiasis, the action of both components associated with the innate immune response and the activity of a number of interrelated virulence factors of yeast-like fungi is necessary.

## 6. Summary

The above-listed factors and mechanisms are not the only ones currently being investigated in the context of VVC pathogenesis. In addition to the enormous complexity of molecular and immunological processes involved in the described phenomena and the difficulties associated with determining the specific functions of individual components, both related to *Candida* and the host’s immune system, problems also arise with ensuring reliable experiments in the research laboratory. The tools most commonly used to assess fungus-host interactions are mouse models and, less frequently, rat models. Many of the discussed mechanisms were observed in an animal model and then confirmed in studies using clinical material taken from female volunteers. However, there are many more laboratory observations, mainly regarding mice, waiting to be verified in human studies. However, the proper and reliable design of such studies continues to be difficult. A similar situation applies to experiments using reconstituted vaginal epithelial cells. Despite the above, recent years have undoubtedly brought many milestones in understanding the pathomechanism of the development of VVC. Although there is still a long way to go to understand the development of VVC fully, the identified factors and mechanisms are already the goal of new antifungal therapies under development, aimed both at fighting an active infection and preventing its development.

## Figures and Tables

**Table 1:** The most important virulence factors associated with *Candida* biofilm formation during VVC and their functions.

Factor	Genes	*Candida* Strains	Function in Biofilm Formation during VVC	References
Als proteins	family of *ALS*	*C. albicans* C. *parapsilosis* *C. tropicalis* *C. dubliniensis* *C. guillermondii* *C. lusitaniae*	adhesion to VECs	[8,42,47,48]
Epa protein	*EPA*	*C. albicans* *C. glabrata*	adhesion to VECs	[8,42,51]
Hwp1 protein	*HWP1*	*Candida spp.*	adhesion to VECs	[8,41,44,48]
Sap enzymes	family of *SAP*	*C. albicans* *C. parapsilosis* *C. tropicalis* *C. dubliniensis*	adhesion to VECs, phenotypic switching, formation of hyphae and its adhesion and penetration into VECs	[24,42,47,50,54]
yapsins	*YPS*	*C. glabrata*	adhesion to VECs, phenotypic switching, formation of hyphae and its adhesion and penetration into VEC	[8,53,54]
lipases	*LIP*	*C. albicans* *C. parapsilosis* *C. tropicalis*	increasing adhesion to VECs	[42,50,53,59]

## Data Availability

Not applicable.
